# Electrostatic Variation of Haemagglutinin as a Hallmark of the Evolution of Avian Influenza Viruses

**DOI:** 10.1038/s41598-018-20225-3

**Published:** 2018-01-31

**Authors:** Alireza Heidari, Irene Righetto, Francesco Filippini

**Affiliations:** 1 Department of Comparative Biomedicine and Food Science, University of Padua, viale dell’Università 16, 35020 Legnaro, (PD) Italy; 2 Department of Biology, University of Padua, via U. Bassi 58/B, 35131 Padova, Italy

## Abstract

Avian influenza virus is a zoonotic agent that significantly impacts public health and the poultry industry. Monitoring viral evolution and spread is crucial for surveillance and tracing programmes, which are currently based on serological or DNA sequencing-phylogenetics analysis. However, virus-host interactions, antigenic drift and spreading of viral clades strongly depend on variation in the surface features of capsid proteins. We report here that *in silico* comparative structural analysis of haemagglutinin can reveal relevant evolutionary fingerprints, particularly when integrated with sequence-based analyses. Phylogenetic analyses of H9 viral strains from wild birds and poultry, performed with different methods, reliably led to clustering of viruses into five main groups. Subsequent comparison of structural features showed congruence between such clustering and surface electrostatic fingerprints. These latter fingerprints relate group-specific variations in electrostatic charges and isocontours to well-known haemagglutinin sites involved in the modulation of immune escape and host specificity. This work suggests that the integration of structural and sequence comparisons may enhance investigations of trends and relevant mechanisms in viral evolution.

## Introduction

Wild waterfowl are the primary reservoirs of avian influenza (AI) viruses, which can also sporadically infect domestic birds and mammalian/human hosts^1^. Therefore, setting up a coordinated global surveillance network and studying viral evolution is crucial for monitoring genetic changes and predicting ‘evolutionary trends’, especially when considering viral clades for which avian to mammalian/human host switching has been reported^2^. The risk for human/animal health also depends on the emergence of novel reassortant viruses, especially where multiple strains and clades co-circulate^3^. H5N1 AI viruses are unique in their ecological success, broad host range and geographical spread^4^; however, recently reassorted subtypes (H7N9, H9N2, and H10N8) may also jump the host-species barrier, increasing concerns regarding their pandemic risk^5^. Studying virus variation possibly related to the low-pathogenicity (LPAI) to high pathogenicity (HPAI) shift and antigenic drift is tremendously relevant to human/animal health, the poultry industry, and vaccine efficacy. To date, H5 and H7 AI viruses have been reported to evolve from an LPAI to HPAI form after their introduction into poultry from wild bird reservoirs^6^, and H9N2 viruses have occasionally been transmitted from poultry to mammals/humans. Therefore, H5, H7, and H9 viruses are intensively studied as top potential pandemic agents^7,8^. Current AI vaccines are based on the elicitation of a neutralising antibody (Ab) response against the major haemagglutinin (HA) epitopes^9^. HA – the main viral surface antigen - plays a central role in AI virus evolution by mediating attachment and penetration into the host cell; mutations in HA immune-dominant regions may result in antigenic drift, allowing the virus to escape Ab neutralisation^9^. In H5N1 viruses, charge redistribution at the surface of the Receptor-Binding Domain (RBD) sub-region of HA relates to the branching of still-circulating clades relative to no longer circulating ones^10^. To assess whether electrostatic variation is a general fingerprint of AI virus evolution, we screened a large dataset of H9 viruses. Comparative analysis of groups and clades confirmed congruence between phylogenetic and electrostatic clustering, suggesting electrostatic variations as widely applicable fingerprints of avian influenza viral evolution.

## Results

### Phylogenetic Clustering of AI H9 HA

We used genetic correlation to follow objective criteria to properly sort strains based on their phylogenetic topology and on specific evolutionary distances that reflect the diversity of the AI H9N2 subtype. A dataset and alignment of 1669 HA sequences were subjected to phylogenetic tree reconstruction using the neighbour-joining (NJ), maximum-likelihood (ML), and Bayesian methods. Independent of the algorithm used, i.e., NJ, ML or Bayesian, H9N2 viruses reliably sorted into 5 different monophyletic groups (referred to as A, B, C, D and E) consisting of 28 total clades. Such clades - identified by numbers - were separated based on an inter-clade average distance ≥5% and an intra-clade average distance <5%; separation for each identified clade was confirmed by a C-value ≥ 1. For the sake of clarity and disambiguation, in this context, the C-value does not refer to DNA content/genome size; it instead represents a ratio as determined by the LABEL method used for H9N2 clade partitioning^11^. Specifically, it is the ratio of the average pairwise distance between a particular taxon and its closest neighbouring group divided by the average pairwise distance within that selected clade. C-values ≥1 indicate that clades differ from each other, while clades with C-values <1 should be grouped together^11^. Groups and clades were assigned when at least three isolates with different epidemiological histories formed a distinct taxonomic group with bootstrap values at the defining node ≥60%. Clade separation based on distance value cut-offs was confirmed using two different calculation algorithms, as described in the methods section. Such A to E phylogenetic grouping was further confirmed using a ‘seed’ alignment with 360 HA sequences, and 28 representative viruses (one from each clade) were selected for subsequent structural bioinformatics analyses. For space constraints, both the seed tree and a table with details concerning the representative viruses are presented in the Supplementary file. Further work is necessary to provide a comprehensive ‘classification’ for H9N2; this is not the aim of this work, which instead focuses on structural feature comparison.

### Clustering by Electrostatic Distance for AI H9 viruses: Heatmaps

**Figure 1:**
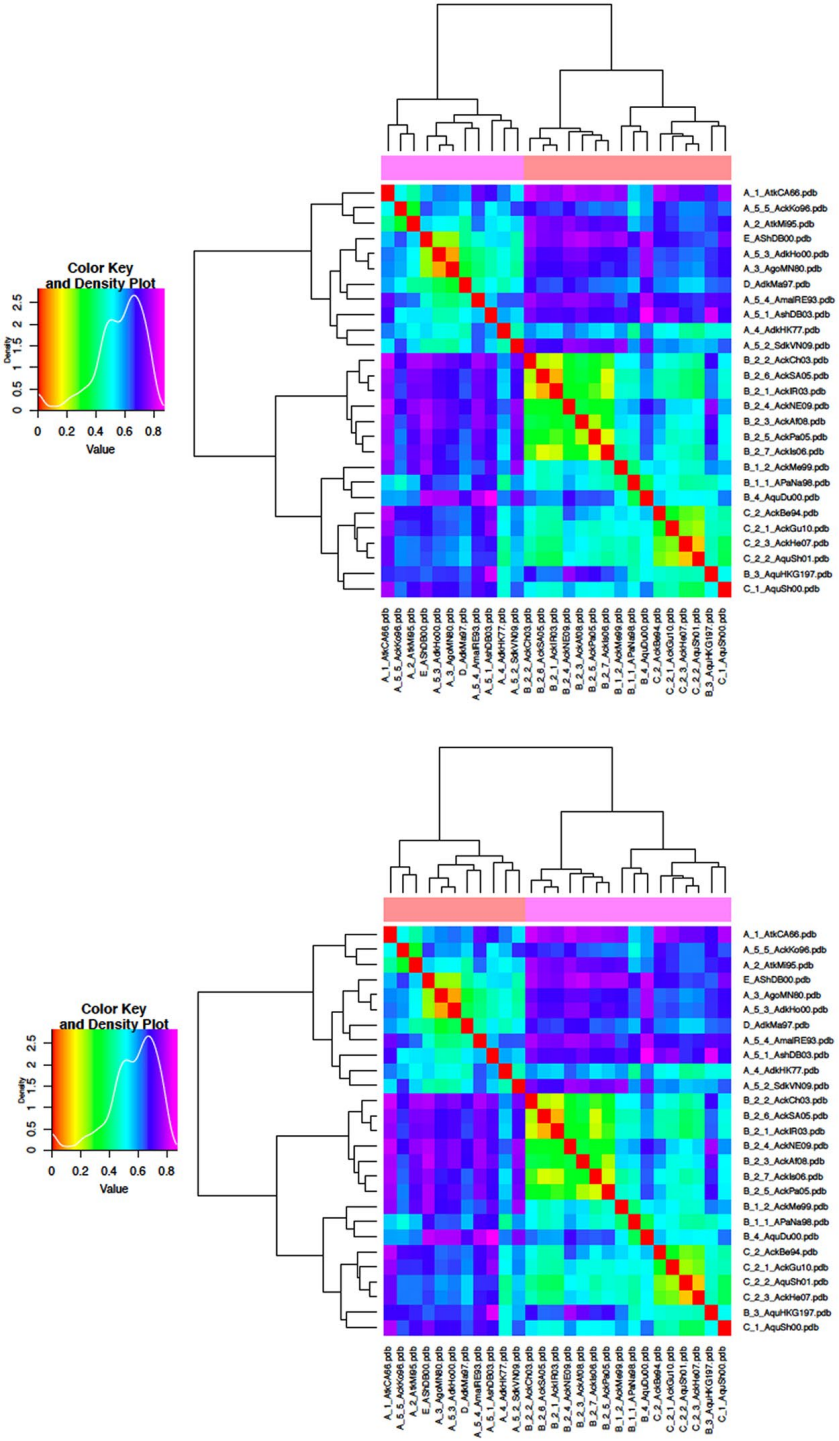
Clustering of representative H9N2 HA RBDs by ED. Density plots (left side) and heatmaps (right side) were calculated using both the Carbo (upper panel) and Hodgkin (lower panel) indexes. Warm (red) to cold (violet) colour transition corresponds to increasing ED.

A preliminary report described the use of H5N1-related surface electrostatics for clade evolution and spread^10^; we report here that clustering of H9 viruses by electrostatic distance (ED) also shows substantial agreement with phylogenetic grouping, suggesting this as a general hallmark of AI virus evolution. Given that the tool for electrostatic analysis accepts as input data only Protein Data Bank (PDB) files (rather than aa sequences), each H9 RBD target sequence was modelled via homology modelling (see methods). Indeed, all target sequences showed high identity with the template, thus resulting in very low Root Mean Square Deviation (RMSD) values, i.e., very high structural closeness. However, understanding the precise side chain orientation is crucial to properly performing any surface feature analysis. Therefore, all models underwent refinement and quality analysis (see methods). Semi-quantitative ED evaluation and clustering of the spatial distributions of the electrostatic potentials were obtained by WebPIPSA (Protein Interaction Property Similarity Analysis)^12^. Heatmaps (Fig. 1) showed that the ED among H9 representative viruses from ‘wild bird’ groups A+D+E and those from ‘poultry bird’ groups B+C was high (dark blue, violet or magenta colours, as coded in density plots). In contrast, the prevalence of light blue, green and yellow colours highlights a lower distance between A and D (or A and E or D and E), as well as between B and C. The only exception to the overall substantial agreement of electrostatic clustering with phylogenetic grouping is represented by clades B3 and B4 (both isolated from the same host bird - quail), which are closer to C2 than to B2 strains. No meaningful difference in heatmaps was observed when using either Hodgkin or Carbo indexes (Fig. 1).

### Variation in Electrostatic and Hydrophobicity Features Among AI H9 Groups and Clades

**Figure 2:**
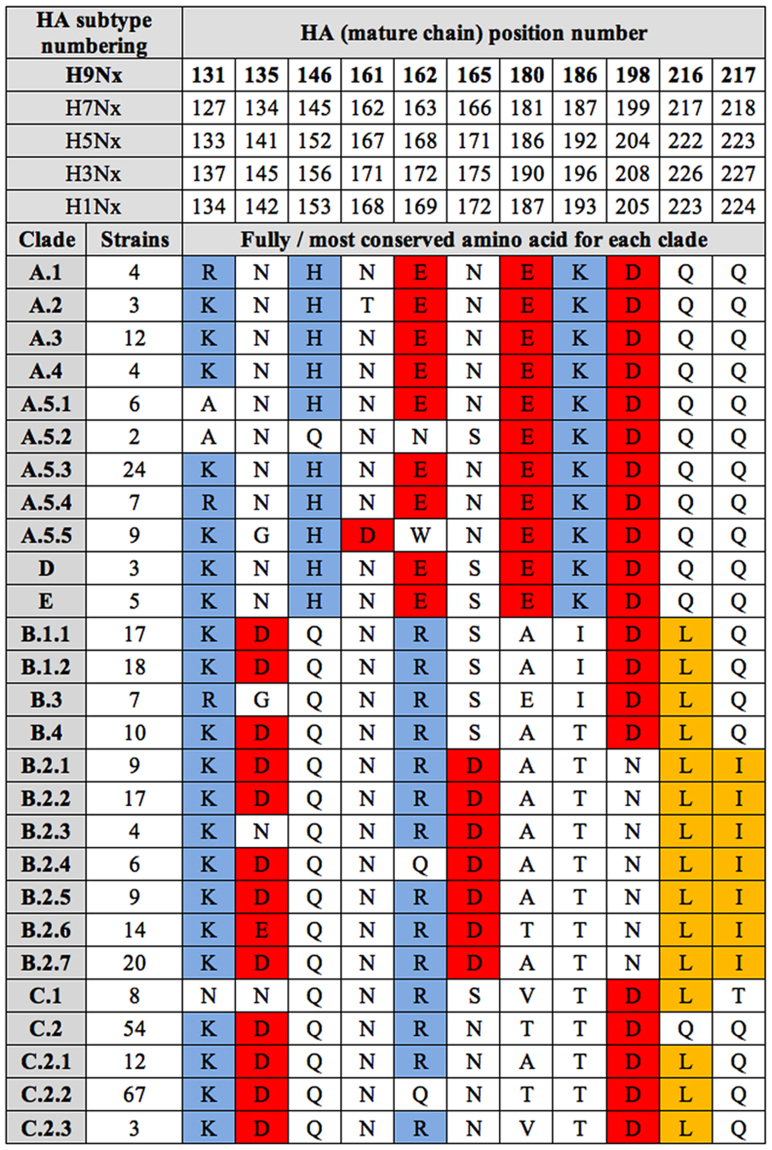
Distribution of negatively (red) and positively (blue) charged and hydrophobic (yellow) residues at the HA RBD among representative H9N2 viruses.

In-depth analysis of charged residue distributions, including their variation in number and position, and isocontours in HA further confirmed that their variation, specifically concerning the RBD sub-region and suggested electrostatic fingerprints, does relate to different H9 groups. For the readers’ convenience, Fig. 2 compares the most recently published HA mature chain numberings for H1, H3, H5, H7 and H9^13^, and it highlights group- and sub-group-specific variations within the RBD. In particular, group-associated ‘charge redistribution’ was observed at RBD positions 135, 146 and 162 (H9 numbering): the net charge for these positions is zero in all groups (reflecting the sum of two opposite charges and a neutral residue). However, the charge distribution pattern shared by ‘wild bird’ groups A+D+E was neutral-positive-negative, whereas it was negative-neutral-positive in ‘poultry’ groups B+C. RBD compensatory mutations appear to maintain group-specific fingerprints and net charges while progressively ‘sliding’ charges over RBD sites in the viral population. At position 135, almost all (94%) Class A viruses shared an uncharged residue, predominantly (72%) Asn, which was 100% conserved in classes D+E; mutation to a charged residue (N135D) was only found in 6% of viruses from clades A.5.3, A.5.4 and A.5.5. Instead, negativisation at position 135 was most often observed in both class B (85%) and C (92%), with a predominance of Asp/Glu over other amino acids in almost all B+C clades. A compensatory mechanism was observed for exceptions, i.e., for clades not sharing a negative charge at 135. For example, clade C.1 lacked the negative charge of classes B+C and instead shared (100% sampled viruses) N135 with classes A+D+E; however, this was compensated for as C.1 is also the only B+C clade missing a positive charge at position 131. Similarly, B.3 (showing a preference for Gl35) is also the only B+C clade with a negative charge (Glu) replacing an uncharged residue at position 180. Residue 146 is His in 95% of class A viruses and in all D+E strains, and Gln in almost all B (>99%) and C (98%) clades. The only class A exception was clade A.5.2, with Q146 (such as B+C) instead of H146 (common to A+D+E). However, once again, a counterbalancing event was observed: depositivisation at position 146 of A.5.2 was compensated for by specific denegativisation at position 162 (E162N). Loss of a negative charge at E162 (otherwise shared by A+D+E groups) was also seen in clade A.5.5 (E162W in 100% of viruses); however, intriguingly, the lost negative charge was rescued at the contiguous amino acid position by the equally conserved (100%) and peculiar mutation N161D. A negative charge at position 162 (or 161) is thus a landmark for the A+D+E groups. In the B+C groups, major residues at 162 are Arg and Gln, with the predominance of the former over the latter in all clades but B.2.4, where the opposite predominance was observed. Therefore, ongoing positivisation of position 162 appears to also be a landmark for viruses circulating in poultry. Greater inspection at position 217 showed a meaningful difference with respect to position 216. In addition to A+D+E groups (>99% strains), the ‘original’ Gln was also highly conserved in group C (82% strains), and in C.1 the major residue was any polar residue (Thr, in 97% of strains). Instead, Gln was 100% conserved in clades B.1.1, B.1.2, and B.3, whereas a polar to hydrophobic transition was ongoing in clade B.4 (Gln, however, was still the major residue) and fully fixed (100%) in the whole B.2.x sub-group (sharing Ile as a major residue). Altogether, counterbalancing mutations observed at positions 131–135 (C.1), 135–180 (B.3), 146–162 (A.5.2) and 161–162 (A.5.5) appeared to support a compensatory mechanism for maintaining the overall net charge while sliding charges over the RBD itself, i.e., for keeping group specific landmarks along with the contemporary creation of novel fingerprints. At positions 180–186, the net charge was zero in all H9 clades but B.3, where A180E was indeed compensatory for D135G. In A+D+E viruses, neutral charge results from the sum of opposites (+1 −1 = 0), while in B+C viruses, both charges are replaced by neutral residues (0 + 0 = 0), thus maintaining the net charge. Specific variation also concerned positions 216–217: at 216, A+D+E clades share a highly conserved (>99% strains) polar residue (Gln), while most (86%) B+C viruses showed a polar to hydrophobic Q216L transition. Instead, Gln is still the major residue in clades B.1.2 and C.2. At position 217, sub-group variation was observed: only B.2.x viruses shared a hydrophobic residue (Ile), while Gln was common to all other A+B+C+D+E clades. Such sub-group specific variation was not limited to hydrophobic patches, as ‘charge sliding’ also occurred between position s165 and 198: H9 clades are negatively charged at 198, except for B.2.x viruses, which show a polar residue (primarily Asn). Such ‘denegativisation’ was compensated, however, in B.2.x by an equally peculiar acquisition of a negative charge at 165, where Asp is 100% conserved.

### Residues Involved in Changes at the H9N2 RBD are Surface-Exposed

**Figure 3:**
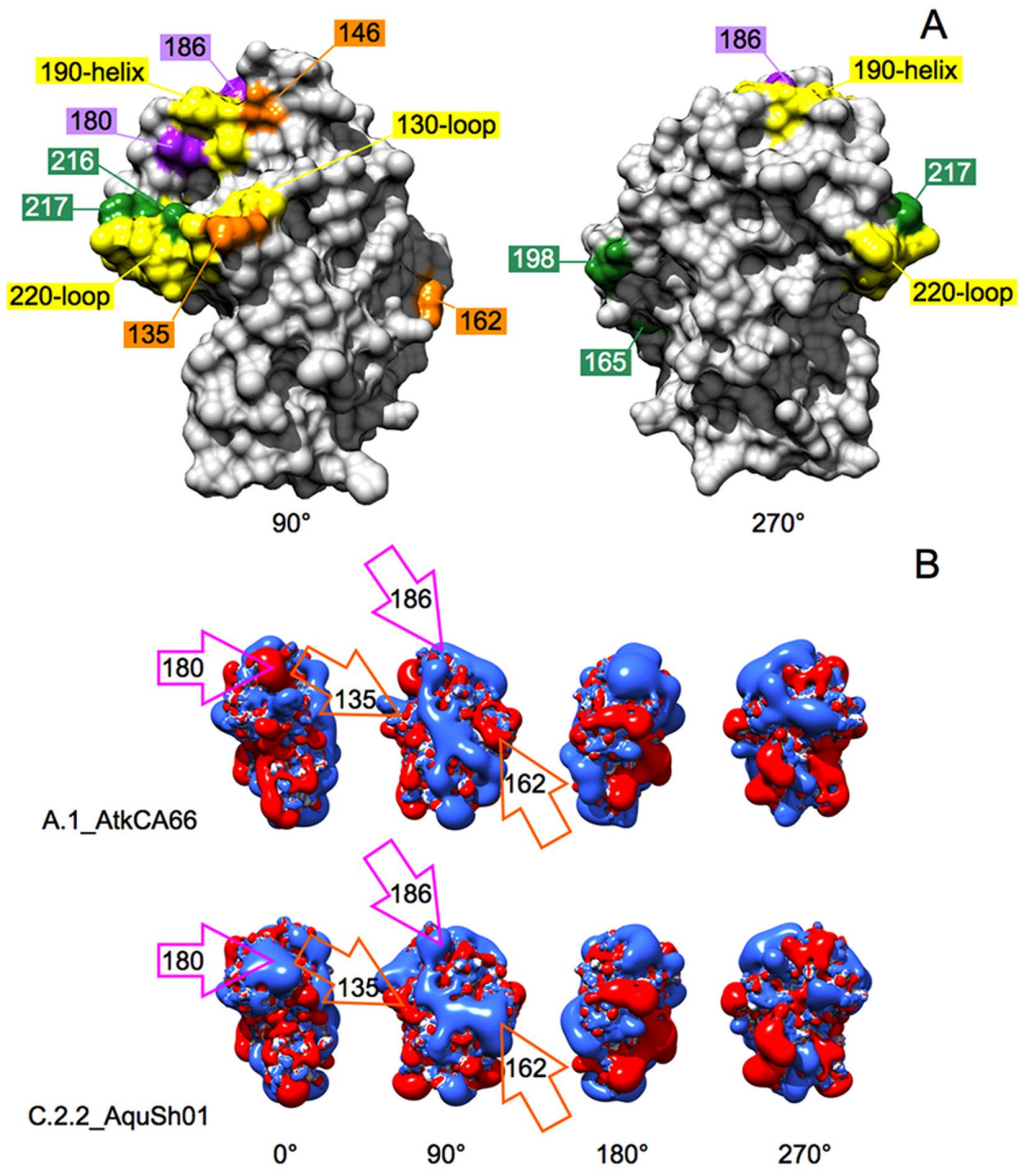
Upper panel: 90° and 270° views of the RBD sub-region of H9N2 HA highlighting relevant surface epitopes. Lower panel: four-step 90° views of representative H9N2 RBDs highlighting ED mediated by mutation of specific residues.

The RBD from the solved structure of the H9 HA was viewed to highlight the nine positions involved in group or sub-group specific variation. In the top of Fig. 3, the RBD surface is coloured grey and antigenic sub-regions mediating SA binding (130-loop, 190-helix and 220-loop) are highlighted in yellow. The isopotential contours of the viral strains A.1_AtkCA66 and C.2.2_AquSh01, representative of electrostatic fingerprints from ‘wild bird’ A+D+E viruses and ‘poultry’ B+C viruses, are shown in the lower panel of Fig. 3. Residues 135, 146 and 162, which are involved in group-specific ‘charge redistribution’, are surface exposed (orange); in particular, 146 is close to the 190-helix and 135 is part of the 130-loop. Positions 180–186 (mediating the ‘charge loss’ observed in the A+D+E to B+C transition) are surface-exposed as well (purple) and are part of the 190-helix. The four positions involved in group and sub-group variation are also surface-exposed (green); 216 and 217 are part of the 220-loop, while 165 and 198 protrude from the other RBD ‘side’. Finally, 131 and 161, involved in compensatory variation (Fig. 2), were also confirmed as surface-exposed (not shown). Therefore, a deeper view of the surface variation among H9 groups, sub-groups and clades could be obtained via electrostatic analysis of RBD models refined for side chain orientations. Both strain A.1_AtkCA66 and C.2.2_AquSh01 matched in all position patterns (Fig. 2) typical for A+D+E or B+C, respectively. At 162, they clearly showed opposite charges; at 135, the expected contours were found again, as A.1_AtkCA66 showed no charge while C.2.2_AquSh01 showed a negative protrusion in the corresponding area. Comparative analysis at 180–186 showed that the loss of both charged residues in the A+D+E to B+C transition did not result in ‘neutralisation’ of the corresponding surface area. Instead of just an expected negative (Glu)-to-neutral (Thr) shift, a seeming ‘positivisation’ (increased blue area) was observed at position 180 in C.2.2_AquSh01, depending on the enlargement of neighbouring electrostatic contours. For completeness, the isopotential contours of the RBDs from all 28 representative strains used for creating heatmaps are presented in the Supplementary file.

## Discussion

‘Classic’ studies on AI virus evolution are based on antigenic and phylogenetic analyses^14^. Recently, fingerprints for H5N1 evolution and spread were obtained via deep analysis of the HA RBD surface; when comparing RBD structures, H5 was found to be much closer to H9 than H3 and H7 and, when comparing electrostatics, even closer to H9 (from a different phylogenetic group) than to H2 (same group)^10^. This prompted us to investigate whether similar mechanisms might underlie H5 and H9 evolution and spread. Indeed, this work indicates that the H9 HA gene has a remarkable similarity to H5 circulation and evolution. AI H9 strains show extended branches as these viruses continue to co-circulate in different regions and host species, and this allows the clades to further evolve and differentiate. Such H5-H9 structural agreement suggests RBD ‘charge redistribution’ as a general landmark for AI virus evolution and spread. Intriguingly, most of the changes observed in H9 RBD occur at sub-regions crucial to sialic acid (SA) binding, host specificity and immune escape/antigenic drift^15^: the 130-loop (129–132), 190-helix (180–188) and 220-loop (211–218). Indeed, positions 162 and 217 are both involved in immune escape^16^ and in group-specific charge redistribution: ‘denegativisation’ at 162 in two clades from group A is compensated by either ‘depositivisation’ at 146 (in A.5.2) or ‘negativisation’ at 161 (in A.5.5). A sub-group-specific, polar to hydrophobic transition occurs instead at 217 and is likely involved (as 220-loop) in increased binding to 〈2–6 SA and thus in improved affinity for the human host. Residue 224 in H1N1 (corresponding to 217 in H9) mediates hydrogen bond interactions with 〈2,6-SA^17^; the involvement of H9 217 in host range modulation is also based on studies of H5N1^18^. H3 227 (corresponding to H9 217) is located between H3 226 and 228, playing a key role (as 220-loop) in host range restriction^19^. In this work, group-specific variation in H9 216 (corresponding to H3 226) was also observed. In H9 180–186 (190-helix) the conserved dual opposite-charge pair in groups A+D+E shift to an uncharged pair in B+C. Contemporary loss of the two opposite charges is somehow ‘compensatory’ for the original RBD net charge. It is noteworthy that in AI viruses, mutations increasing or decreasing the charge of the SA binding RBD region can modulate binding avidity and affinity, and thus frequently observed charge counterbalancing is likely to compensate for gain and loss effects, thereby maintaining the HA-NA charge balance^15^. However, in an A+D+E to B+C group transition, compensation only maintains the net charge, while the overall loss of two charged residues from the RBD in B+C viruses occurs. This, in turn, is likely to favour immune escape because of both the location of the two residues and the well-known role of charged amino acids in modulating protein antigenicity and immunogenicity. In fact, it is well-known that both positively and negatively charged residues improve the antigenic recognition (up to several fold, depending on their number in the antigenic site) by creating further salt bridges with the recognising Ab complementary surface^20^. Charge variation also occurs at position 146 (involved with 135 and 162 in charge redistribution), which is exposed at the RBD surface close to the 190-helix (Fig. 2). Therefore, changes such as the observed ‘depositivisation’ at position 146 might influence the SA binding affinity and specificity. Considering that chickens possess both 〈−2′3′ and 〈−2′6′ SA receptors, it is tempting to speculate that such changes could be linked to host adaptation and species specificity^21^. Sequence comparison was able to infer group- and sub-group-specific fingerprints, which are presented in Fig. 2 as sequence patterns; however, once sequence analysis was complemented by the structural bioinformatics approach, a picture of ongoing molecular evolution arises. Comparison of the electrostatic isocontours unveiled combined mutations resulting in modulation of relevant surface features by altering the local equilibrium in surrounding areas (e.g., salt bridges or repulsions, hydrophobicity changes, and decreased or increased charge density). Even though further work is needed to fully clarify the complex network of equilibria that can be altered by specific mutations, evidence from this study supports the integration of current phylogenetic analyses with sequence-based and structural investigations of surface features as a front-end strategy for inferring trends and relevant mechanisms in influenza virus evolution.

## Methods

### Phylogenetic analyses

HA gene nucleotide sequences of H9N2 subtype were retrieved from the NCBI and GISAID (Global Initiative on Sharing Avian Influenza Data) EpiFlu databases (http://www.gisaid.org). Nucleotide sequences at least 1500 bp in length were selected. Multiple sequence alignment of HA sequences was performed with MAFFT version 7 (http://mafft.cbrc.jp/alignment/server). Redundant isolates with 100% sequence similarity (i.e., redundant sequences) were identified and removed, resulting in a final HA dataset and alignment of 1669 sequences that was subjected to phylogenetic tree reconstruction. The NJ, ML, and Bayesian methods were used to construct three different phylogenetic trees for comparison. Analysis of the best-fit substitution model was performed using MEGA5^22^, and the goodness-of-fit of each model was measured by Bayesian Information Criterion and corrected Akaike Information Criterion (AICc). The General Time Reversible (GTR) model with a discrete gamma distribution (+$\wp$) allowing for invariant sites (+I) was selected based on AICc and used in all data analyses. MEGA5 was also used to perform phylogenetic analysis, and the evolutionary history was inferred by both NJ and ML methods^23^, with standard error being calculated based on 1000 bootstrap replicates. Furthermore, PhyML (version 2.4.4)^24^ was used to create ML trees. The GTR + $\wp$ + I model of nucleotide substitution was used for analysis, with an estimated gamma shape parameter. Robustness of the groups was assessed using the bootstrap approach with 100 replicates. The Bayesian phylogenetic tree was inferred using MrBayes software^25^ and applied to generate the dendrograms, as well as to assess statistical support for the branches from the trees generated by the original dataset. For ease of display, and to ensure that the clade topology would be maintained when fewer isolates were used, a small representative dataset of 360 H9N2 HA sequences was created and analysed by the same aforementioned phylogenetic models (seed tree in this work). Phylogenetic trees were visualised using FigTree version 1.3.1 (http://tree.bio.ed.ac.uk/software/figtree/).

### Structural Modelling and Model Refinement

Structural models for the RBD regions of target HA proteins were obtained by homology modelling using SWISS-MODEL^26^ and the PDB 1JSH structure as the H9N2 HA template; such models were then refined using SCWRL, which for optimisation integrates: (i) a backbone-dependent rotamer library, (ii) a simple energy function based on rotamer frequencies and a repulsive steric energy term, as well as (iii) a graph decomposition to solve the combinatorial packing problem^27^. Model quality was then checked via the QMEAN server. QMEAN is a scoring function that measures multiple geometrical aspects of protein structure, ranging in value from 0 to 1 with higher values indicating more reliable models^28^. Protein structures were viewed using UCSF Chimera^29^ v. 1.11.2 (http://www.cgl.ucsf.edu/chimera/).

### Analysis of Electrostatic Potentials

Comparative analysis of electrostatic potentials was performed through the Opal web server connected to the Adaptive Poisson-Boltzmann Solver (APBS) server (http://www.poissonboltzmann.org/apbs), calculating the spatial distribution of the electrostatic potential at physiological ionic strength (I) = 150 mM, assuming +1/−1 charges for the counter-ions. Partial charges and van der Waals radii were assigned via PDB2PQR according to the PARSE force field^30^, choosing an interior Σ_p_ = 2 and Σ_s_ = 78.5 for protein and solvent, respectively. Contouring was done at ±1k_B_T/e and viewed using UCSF Chimera. ED was calculated with Hodgkin and Carbo indexes at the WebPIPSA server (http://pipsa.eml.org/pipsa). ED between molecules a and b was provided by Eq. (1), where the Similarity Index (SI) was provided in turn by Eq. (2):

1 $$E{D}_{a,b}=\sqrt{2-2S{I}_{a,b}}\,$$

2 $$S{I}_{a,b}=\,\frac{2({p}_{a},\,{p}_{b})}{({p}_{a},\,{p}_{a})+(p{}_{b},\,{p}_{b})}$$

where (*p*_*a*_, *p*_*b*_), (*p*_*a*_, *p*_*a*_), (*p*_*b*_, *p*_*b*_) are the scalar products of the electrostatic potentials over the region where the potentials are compared^12^.

Rigid-body superposition was performed and RMSD was computed using UCSF Chimera.

## Electronic supplementary material

Supplementary information

## References

[1] Nelson MI, Vincent AL (2015). Reverse zoonosis of influenza to swine: new perspectives on the human-animal interface. Trends Microbiol..

[2] Al-Tawfiq JA (2014). Surveillance for emerging respiratory viruses. Lancet Infect. Dis..

[3] Su S (2015). Epidemiology, Evolution, and Recent Outbreaks of Avian Influenza Virus in China. J. Virol..

[4] Guan Y, Smith GJ (2013). The emergence and diversification of panzootic H5N1 influenza viruses. Virus Res..

[5] Trombetta C, Piccirella S, Perini D, Kistner O, Montomoli E (2015). Emerging Influenza Strains in the Last Two Decades: A Threat of a New Pandemic?. Vaccines (Basel).

[6] Alexander DJ (2007). An overview of the epidemiology of avian influenza. Vaccine.

[7] Lin YP (2000). Avian-to-human transmission of H9N2 subtype influenza A viruses: relationship between H9N2 and H5N1 human isolates. Proc. Natl. Acad. Sci. USA.

[8] Butt KM (2005). Human infection with an avian H9N2 influenza A virus in Hong Kong. in. J. Clin. Microbiol..

[9] Velkov T (2013). The antigenic architecture of the hemagglutinin of influenza H5N1 viruses. Mol. Immunol..

[10] Righetto I, Milani A, Cattoli G, Filippini F (2014). Comparative structural analysis of haemagglutinin proteins from type A influenza viruses: conserved and variable features. BMC Bioinformatics.

[11] Shepard SS (2014). LABEL: fast and accurate lineage assignment with assessment of H5N1 and H9N2 influenza A hemagglutinins. PLoS One.

[12] Richter, S., Wenzel, A., Stein, M., Gabdoulline, R. R. & Wade, R. WebPIPSA: a web server for the comparison of protein interaction properties. *Nucleic Acid Res*. **36**(Web Server Issue), W276–W280 (2008). 10.1093/nar/gkn181 PMC2447742 18420653

[13] Burke DF, Smith DJ (2014). A recommended numbering scheme for influenza A HA subtypes. PLoS One.

[14] Stanekova Z, Vareckova E (2010). Conserved epitopes of influenza A virus inducing protective immunity and their prospects for universal vaccine development. Virol. J..

[15] Kobayashi Y, Suzuki Y (2012). Compensatory evolution of net-charge in influenza A virus hemagglutinin. PLoS One.

[16] Peacock T (2016). Antigenic mapping of an H9N2 avian influenza virus reveals two discrete antigenic sites and a novel mechanism of immune escape. Sci. Rep..

[17] Chutinimitkul S (2010). Virulence-associated substitution D222G in the hemagglutinin of 2009 pandemic influenza A(H1N1) virus affects receptor binding. J. Virol..

[18] Gambaryan A (2006). Evolution of the receptor binding phenotype of influenza A (H5) viruses. Virology.

[19] Vines A (1998). The role of influenza A virus hemagglutinin residues 226 and 228 in receptor specificity and host range restriction. J. Virol..

[20] Farber, D. L., Sleasman, J. W. & Virella, G. Immune response: Antigens, Lymphocytes and Accessory Cells. *Medical Immunology*, 6th Edition Chapter 4, pp. 35–54 (2007).

[21] Perez DR (2003). Role of quail in the interspecies transmission of H9 influenza A viruses: molecular changes on HA that correspond to adaptation from ducks to chickens. J. Virol..

[22] Tamura K (2011). MEGA5: molecular evolutionary genetics analysis using maximum likelihood, evolutionary distance, and maximum parsimony methods. Mol. Biol. Evol..

[23] Tamura K, Kumar S (2002). Evolutionary distance estimation under heterogeneous substitution pattern among lineages. Mol. Biol. Evol..

[24] Guindon S, Gascuel O (2003). A simple, fast, and accurate algorithm to estimate large phylogenies by maximum likelihood. Syst. Biol..

[25] Ronquist F, Huelsenbeck JP (2003). MrBayes 3: Bayesian phylogenetic inference under mixed models. Bioinformatics.

[26] Bordoli L (2009). Protein structure homology modeling using SWISS-MODEL workspace. Nat. Protoc..

[27] Krivov GG, Shapovalov MV, Dunbrack RL (2009). Improved prediction of protein side-chain conformations with SCWRL4. Proteins.

[28] Benkert, P., Künzli, M. & Schwede, T. QMEAN server for protein model quality estimation. *Nucleic Acids Res*. **37**(Web Server issue), W510–514 (2009). 10.1093/nar/gkp322 PMC2703985 19429685

[29] Pettersen EF (2004). UCSF Chimera–a visualization system for exploratory research and analysis. J. Comput. Chem..

[30] Sitkoff D, Sharp K, Honig B (1994). Accurate calculation of hydration free energies using macroscopic solvent models. J. Phys. Chem..

