# Supplementary material for: Electrostatic Variation of Haemagglutinin as a Hallmark of the Evolution of Avian Influenza Viruses
Source: Sci Rep. 2018 Jan 31;8:1929. doi: 10.1038/s41598-018-20225-3 (PMC5792503; doi:10.1038/s41598-018-20225-3)
Supplement: Supplementary file 1 — Supplementary information [file 41598_2018_20225_MOESM1_ESM.pdf]

Supplementary Information for manuscript:

Electrostatic Variation of Haemagglutinin as a Hallmark of the Evolution of Avian Influenza Viruses

by Alireza Heidari, Irene Righetto and Francesco Filippini

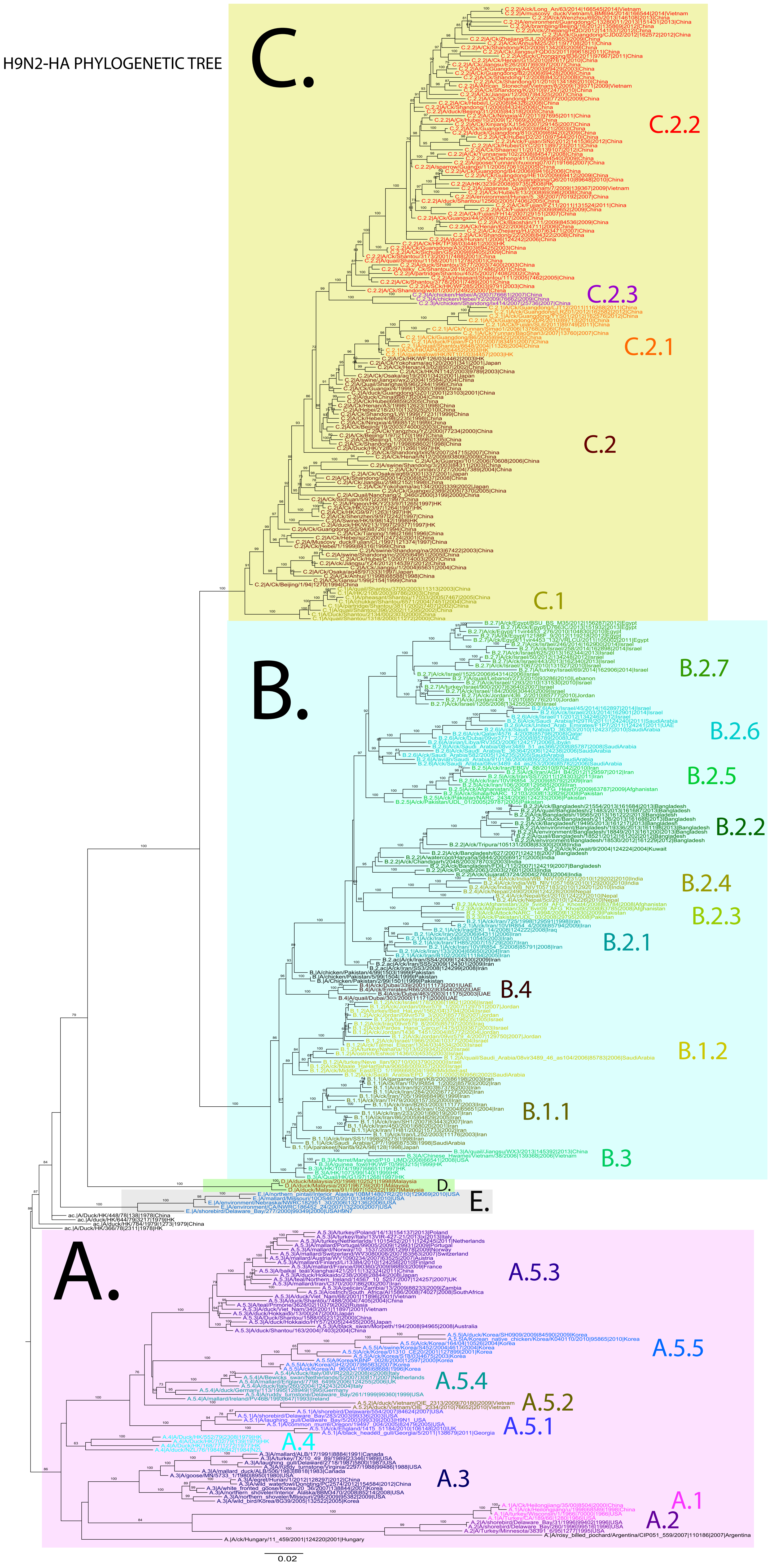

H9N2-HA PHYLOGENETIC TREE
C.
C.2.2
C.2.3
C.2.1
C.2
C.1
B.
B.2.7
B.2.6
B.2.5
B.2.2
B.2.4
B.2.3
B.2.1
B.4
B.1.2
B.1.1
B.3
D.
E.
A.
A.5.3
A.5.5
A.5.4
A.5.2
A.5.1
A.4
A.3
A.1
A.2
0.02

ABOVE: Maximum-likelihood short alignment tree with 360 H9 isolates constructed by PhyML. Groups and clades are colour coded. Estimates of the statistical significance of phylogenies were calculated by performing 100 bootstrap replicates. Numbers in the tree nodes represent the bootstrap support (≥60).

BELOW: 'Representative' viruses (from each group and clade) used for electrostatic analyses.

Group	Clade	Countries	Hosts	Full name	Short name	NCBI AC
A	A.1	USA, China	Avian	A/turkey/CA/189/66	A.1_AtkCA66	AAD49000
	A.2	USA	Avian	A/turkey/Minnesota/38391-6/95	A.2_AtkMi95	AAD48997
	A.3	China, Korea, USA, Canada	Avian	A/goose/MN/5733-1/1980	A.3_AgoMN80	ABB88390
	A.4	Hong Kong, New Zealand	Avian	A/Duck/HK/168/77|1272|1977	A.4_AdkHK77	AF156382.1
	A.5.1	USA, Georgia, UK	Avian	A/shorebird/Delaware Bay/283/2003	A.5.1_AshDB03	AET77176
	A.5.2	Vietnam	Avian	A/duck/Vietnam/OIE_2313/2009|70180|2009	A.5.2_AdkVN09	AB639356.1
	A.5.3	Norway, Vietnam, Iran, China, Italy, Australia, UK, Portugal, Netherlands, Switzerland, France, Finland, Austria, Russia, Japan, South Africa, Zambia	Avian	A/duck/Hokkaido/13/00	A.5.3_AdkHo00	AAQ97383
	A.5.4	Ireland, UK, Italy, Netherlands, Germany, USA	Avian	A/mallard/Ireland/PV46B/1993	A.5.4_AmaIRE93	AB303077
	A.5.5	Korea	Avian Swine	A/chicken/Korea/AI-96004/1996	A.5.5_AckKo96	ACZ48629
D	D	Malaysia	Avian	A/duck/Malaysia/91/1997	D_AdkMa97	AEY75592.1
E	E	USA	Avian envir.	A/shorebird/Delaware_Bay/277/2000	E_AshDB00	AET77024
B	B.1.1	Saudi Arabia, Japan, Iran	Avian	A/parakeet/Narita/92A/98	B.1.1_APaNa98	AB049160
	B.1.2	Lebanon, Israel, Jordan, UAE, Iraq, Saudi Arabia	Avian	A/chicken/Middle East/ED-1/1999	B.1.2_AckME99	GU053201
	B.2.1	Iran, Iraq	Avian	A/chicken/Iran/L248/2003	B.2.1_AckIR03	EF063514
	B.2.2	India, Bangladesh, Kuwait	Avian	A/chicken/Chandigarh/2048/2003	B.2.2_AckCh03	ADL64047
	B.2.3	Afghanistan, Pakistan, Iran	Avian	A/chicken/Afghanistan/329-6vir09-AFG-Khost9/2008	B.2.3_AckAf08	EPI_ISL_63785
	B.2.4	Nepal, India	Avian	A/chicken/Nepal/2490/2009	B.2.4_AckNE09	AFO83282
	B.2.5	Pakistan, Afghanistan, Iran	Avian	A/chicken/Pakistan/UDL-01/2005	B.2.5_AckPA05	ACP50642
	B.2.6	Saudi Arabia, UAE, Qatar, Israel, Libyan	Avian	A/chicken/Saudi Arabia/582/2005	B.2.6_AckSA05	AFO83289
	B.2.7	Israel, Egypt, Lebanon, Jordan	Avian	A/chicken/Israel/1525/2006	B.2.7_AckIS06	ACJ68774
	B.3	Hong Kong, USA, Vietnam	Avian Human	A/Quail/Hong Kong/G1/97	B.3_AquHKG197	AF156378/ AAF00706
	B.4	UAE	Avian	A/quail/Dubai/303/2000	B.4_AquDu00	EF063512/ABM21877
C	C.1	Hong Kong, China	Avian Human	A/quail/Shantou/1318/2000	C.1_AquSh00	EF154910/ABM46230
	C.2	China, Hong Kong, Japan	Avian Swine Human Envir.	A/chicken/Beijing/1/1994	C.2_AckBe94	KF188294/AGO17871
	C.2.1	China	Avian	A/Ck/Guangdong/ZDR/2010|	C.2.1_AckGu10	JF715016.1
	C.2.2	China	Avian Envir.	A/quail/Shantou/1158/2001	C.2.2_AquSh01	EF154916.1
	C.2.3	China	Avian	A/chicken/A/Hebei/2007/76661/2007/China	C.2.3_AckHe07	GQ202056

BELOW: Isopotential contours of the RBDs from all representative H9N2 virus studied in this work Four 90° stepwise rotation views are presented for each representative RBD electrostatic isocontour. Names of the H9N2 virus strains are the same as in table above.

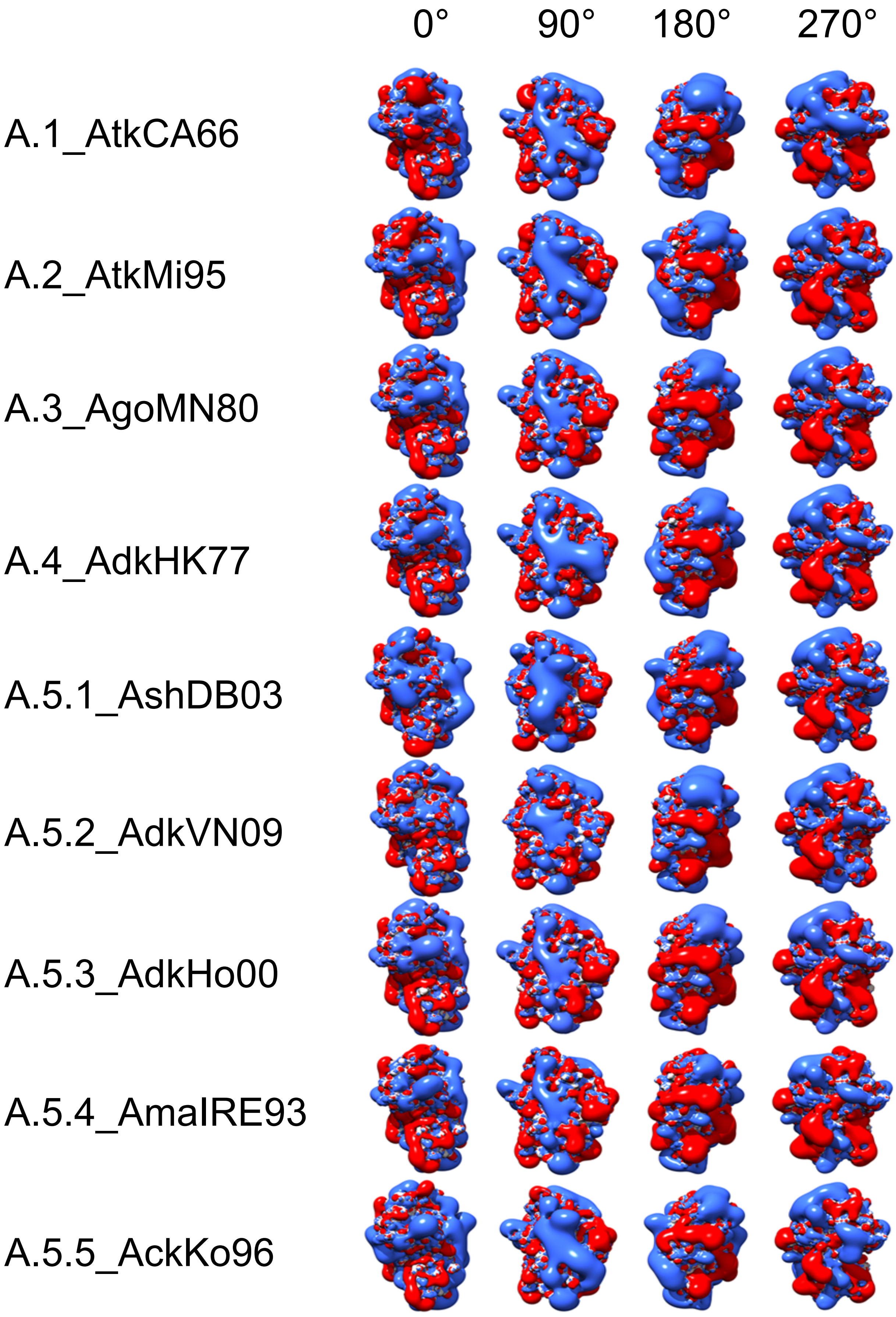
0°
90°
180°
270°
A.1_AtkCA66
A.2_AtkMi95
A.3_AgoMN80
A.4_AdkHK77
A.5.1_AshDB03
A.5.2_AdkVN09
A.5.3_AdkHo00
A.5.4_AmaIRE93
A.5.5_AckKo96

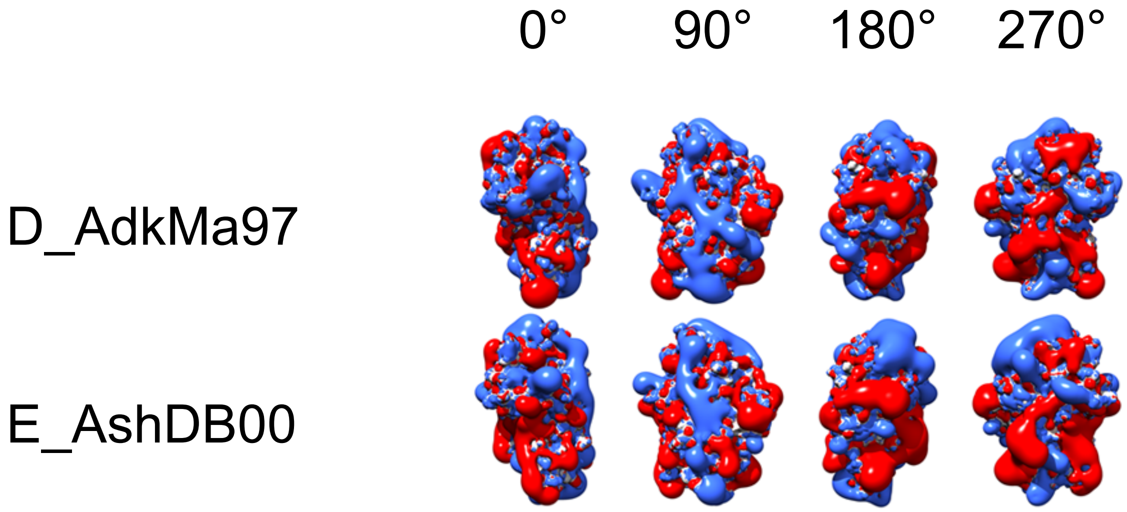
0°
90°
180°
270°
D_AdkMa97
E_AshDB00

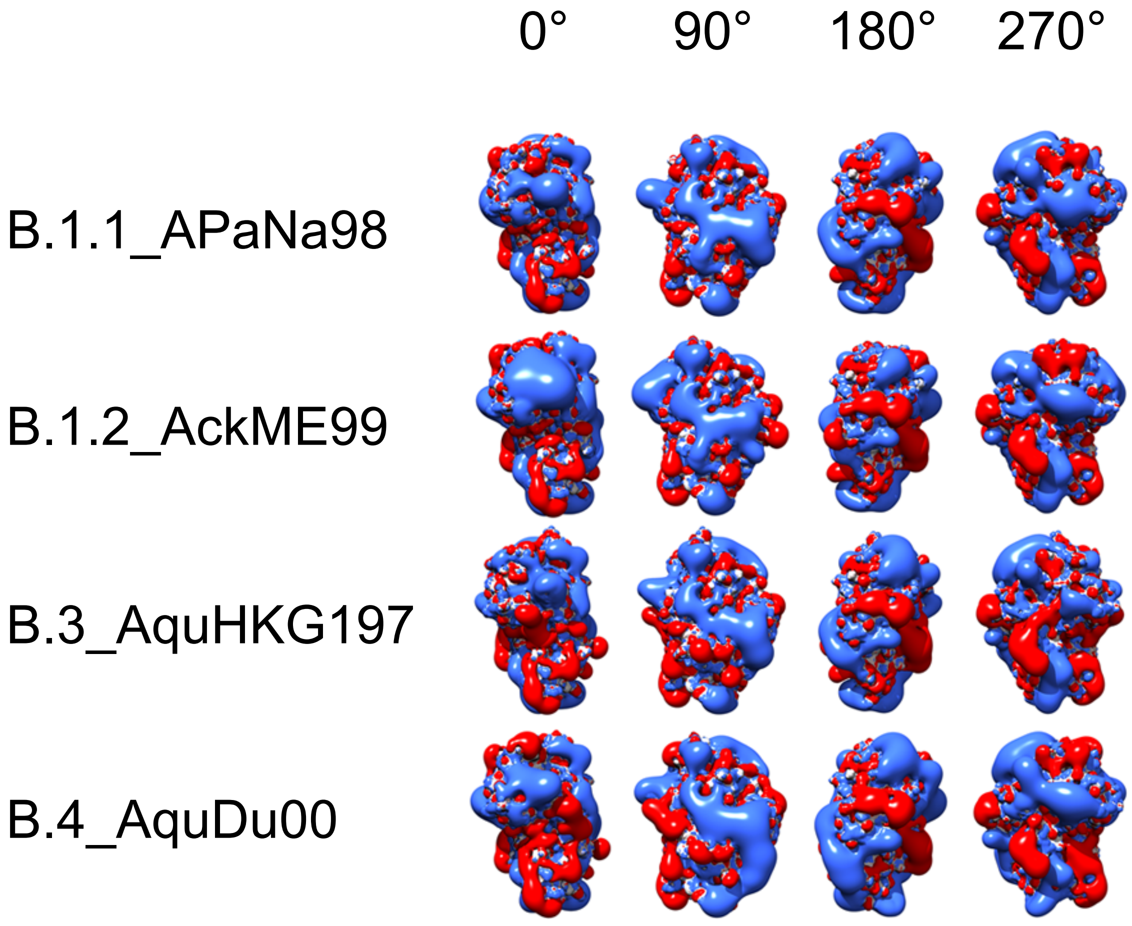
0°
90°
180°
270°
B.1.1_APaNa98
B.1.2_AckME99
B.3_AquHKG197
B.4_AquDu00

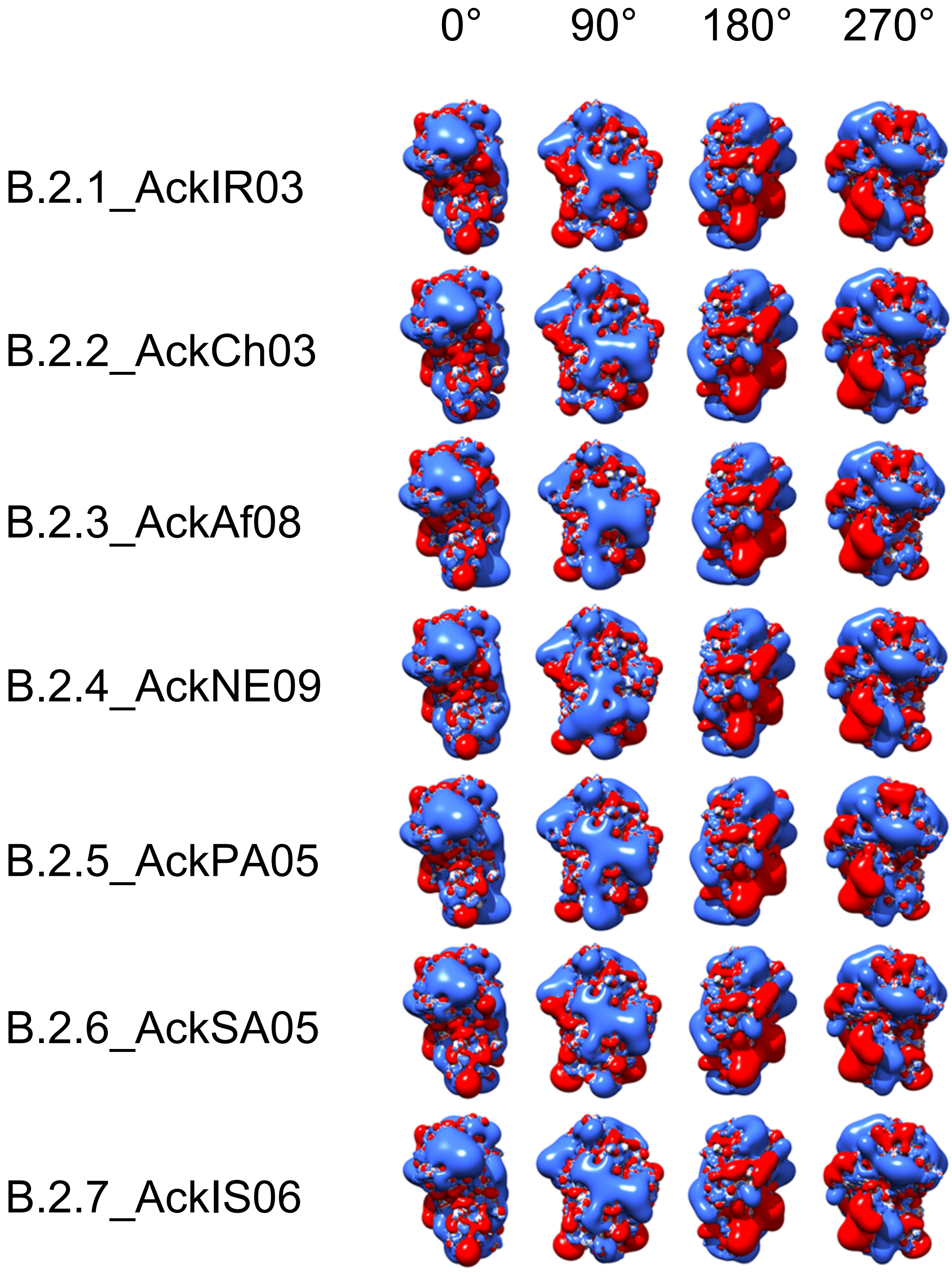

0°
90°
180°
270°
B.2.1_AckIR03
B.2.2_AckCh03
B.2.3_AckAf08
B.2.4_AckNE09
B.2.5_AckPA05
B.2.6_AckSA05
B.2.7_AckIS06

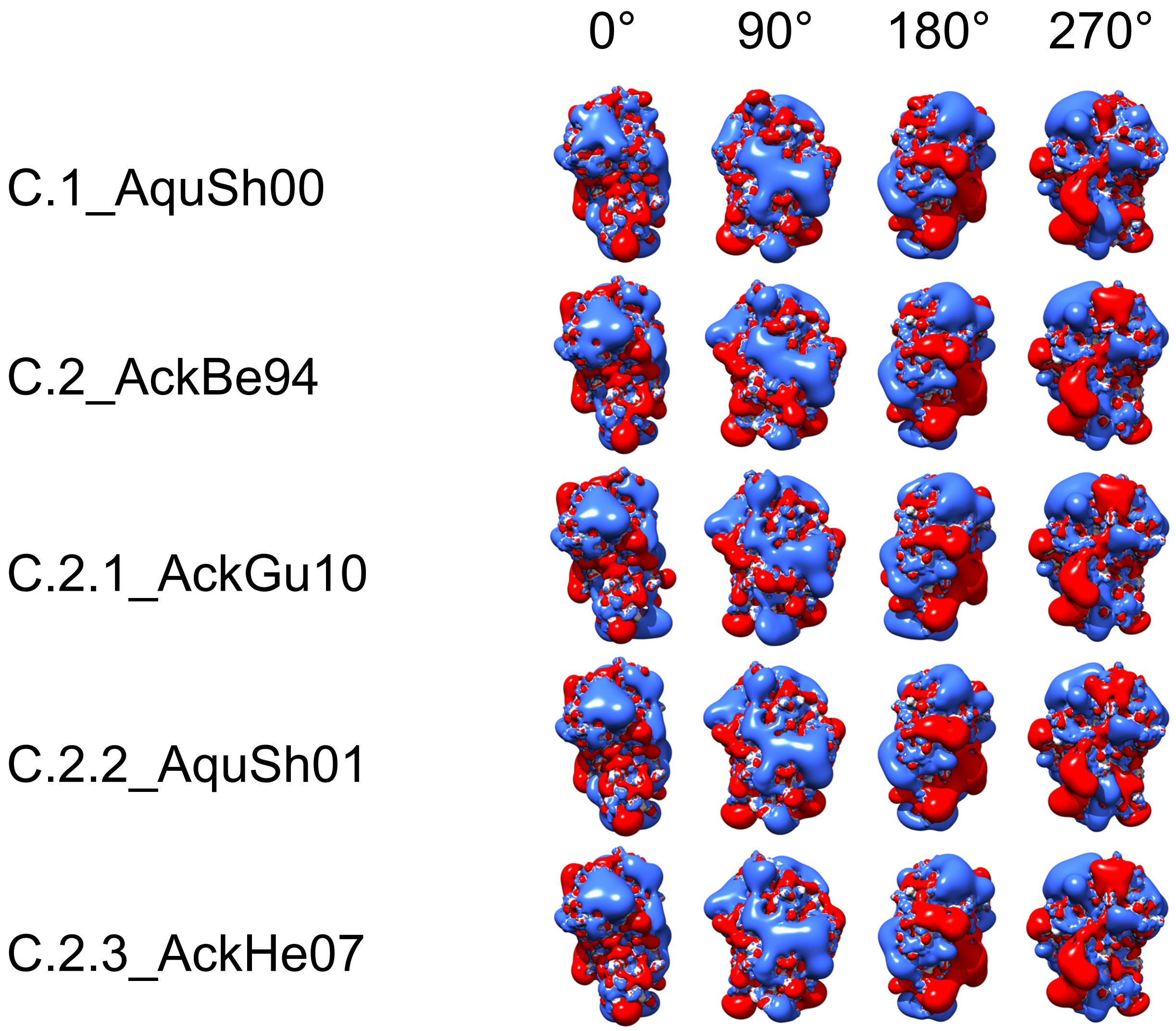
0°
90°
180°
270°
C.1_AquSh00
C.2_AckBe94
C.2.1_AckGu10
C.2.2_AquSh01
C.2.3_AckHe07
